# An improved firefly algorithm with dynamic self-adaptive adjustment

**DOI:** 10.1371/journal.pone.0255951

**Published:** 2021-10-07

**Authors:** Yu Li, Yiran Zhao, Yue Shang, Jingsen Liu

**Affiliations:** 1 Institute of Management Science and Engineering, School of Business, Henan University, Kaifeng, Henan, China; 2 School of Business, Henan University, Kaifeng, Henan, China; 3 Institute of Intelligent Network Systems, Software School, Henan University, Kaifeng, Henan, China; J.C. Bose University of Science and Technology, YMCA, INDIA, INDIA

## Abstract

The firefly algorithm (FA) is proposed as a heuristic algorithm, inspired by natural phenomena. The FA has attracted a lot of attention due to its effectiveness in dealing with various global optimization problems. However, it could easily fall into a local optimal value or suffer from low accuracy when solving high-dimensional optimization problems. To improve the performance of the FA, this paper adds the self-adaptive logarithmic inertia weight to the updating formula of the FA, and proposes the introduction of a minimum attractiveness of a firefly, which greatly improves the convergence speed and balances the global exploration and local exploitation capabilities of FA. Additionally, a step-size decreasing factor is introduced to dynamically adjust the random step-size term. When the dimension of a search is high, the random step-size becomes very small. This strategy enables the FA to explore solution more accurately. This improved FA (LWFA) was evaluated with ten benchmark test functions under different dimensions (D = 10, 30, and 100) and with standard IEEE CEC 2010 benchmark functions. Simulation results show that the performance of improved FA is superior comparing to the standard FA and other algorithms, i.e., particle swarm optimization, the cuckoo search algorithm, the flower pollination algorithm, the sine cosine algorithm, and other modified FA. The LWFA also has high performance and optimal efficiency for a number of optimization problems.

## 1 Introduction

Inspired by various biological systems in nature, many scholars have proposed effective methods that simulate natural evolution to solve complex optimization problems. One of the earliest algorithms was a genetic algorithm proposed by Professor Holland [1]. Researchers then shifted to foraging behaviour of groups of animals, such as the ant colony optimization algorithm (ACO) that simulated the behaviour of ants [2]. Eber proposed particle swarm optimization (PSO) based on bird predation behaviour [3]. In recent years, new heuristic algorithms have been proposed. To name a few, Yang and BED proposed the cuckoo search (CS) algorithm based on the breeding and spawning of cuckoo [4]; The bat algorithm (BA) was proposed based on echolocation behaviour in bats [5]; the whale optimization algorithm (WOA) was inspired by the hunting behaviour of humpback whales [6]; and there is also the grey wolf optimizer algorithm [7], the sine cosine algorithm (SCA) [8], the polar bear optimization algorithm (PBO) [9], etc. More swarm intelligent algorithms were created mainly because of the no free lunch (NFL) theorem [10], which states a single swarm intelligent optimization algorithm cannot solve all optimization problems. The key point of the NFL theorem is that it is meaningless to assess whether an algorithm is good without a real problem, and the performance of an algorithm must be verified by specific problems. Therefore, it is of great significance to study the performance of swarm intelligent algorithms in different application fields [11, 12].

The firefly algorithm (FA) was proposed in 2008 by Xinshe Yang, a Cambridge scholar. The algorithm is based on the luminous characteristics and attractive behaviour of individual fireflies [13]. Compared with other intelligent algorithms, the FA has the advantages of having a simple model, a clear concept, few parameters to adjust, and strong searchability. Like other algorithms, however, it could easily fall into local optimum, resulting in slow convergence speed and low convergence accuracy. As a result, many scholars have made improvements to the standard FA. Yang first introduced the Levy flight into the random part of the location updating formula of the FA and developed an FA with Levy flight characteristics [14]. Subsequently, Yang improved the quality of the FA by introducing chaos into the standard FA and increased the accuracy of the standard FA by dynamically adjusting its parameters [15]. Sharma introduced the inertia weight into the FA; this strategy can overcome the tendency of falling into local optima and can achieve a slow convergence for optimization problems [16]. Farahani and other scholars proposed a Gaussian distribution FA, which referred to an adaptive step size and improved the glow worm algorithm by improving the overall position of the FA population through Gaussian distribution [17]. An FA based on parameter adjustment is better than PSO in solving dynamic optimization problems. Sh. M. Farahani and other scholars introduced an automatic learning machine into the standard FA to adjust the algorithm’s parameters, so that the algorithm can adjust the parameter values at any time according to the environment [18]. Adiland and other scholars improved the self-adaptation of the search mechanism and parameters of individual fireflies and embedded chaotic mapping to solve a mechanical design optimization problem [19]. Carbas also used the FA to solve a steel construction design problem [20]. Tahereh and other scholars used fuzzy coefficients to adjust the parameters of the FA and balanced the local and global search capabilities of the FA [21]. A number of optimization problems of test functions and engineering optimization problems have been verified for performance of the improved FA [22]. The FA is widely used in many fields, especially in computer and engineering fields, such as routine optimization [23, 24], robot path planning [25] and image processing [26–28]. Many scholars around the world have conducted in-depth studies on the theory and application of the FA and are continuously expanding its application fields.

To improve the performance of the FA, this paper proposes an improved FA based on self-adaptive inertia weight logarithmic and dynamic step-size adjust factor (LWFA). Self-adaptive logarithmic inertial weight is also introduced in the updating formula. This strategy can effectively balance the exploration and exploitation capabilities and also improve the convergence speed of the algorithm. A step-size adjust factor is also added into the LWFA to randomly change the algorithm’s step-size, preventing the FA from falling into a local optimum.

Section 2 discusses the standard FA. The proposed LWFA is introduced in Section 3. In Section 4, the complexity analysis and convergence analyses are conducted to calculate the stability and validity of the LWFA. In Section 5, ten benchmark optimization test functions and IEEE CEC2010 test functions are used to evaluate the performance of the proposed algorithm. Experimental function curve results are shown in Section 5. Lastly, the work is summarized in Section 6.

## 2 Standard firefly algorithm

The firefly algorithm (FA) was proposed by a Cambridge scholar Xinshe Yang in 2008 [13]. The FA is a random search algorithm based on swarm intelligence that simulates the attraction mechanism between individual fireflies in nature. To idealize certain characteristics of fireflies when constructing mathematical models of FA, the following idealization criteria are used:

1. Fireflies, both male and female, are attracted only by light intensity between groups regardless of gender;
2. The attraction of fireflies to each other is proportional to the brightness of the light;
3. The brightness of fireflies is related to the objective function value that is to be optimized.

### 2.1 Mathematical description and application

In the firefly algorithm, fireflies have a unique lighting mechanism and behavior, and the light they emit can only be perceived by other individual fireflies within a certain range for two reasons: the light intensity *I* and the distance from the light source *r* are in inverse proportion, and the light can be absorbed by air. The basic principle of the standard FA is that in a randomly distributed firefly population, fireflies with high brightness attract fireflies with low brightness toward them, and each firefly approach a firefly with high absolute brightness in a solution space, update its position, complete an iteration of positions, and find an optimal position to achieve optimization. The brightness of fireflies is related to the objective function value. If fireflies have higher brightness and better positions, they will attract more fireflies. The brightest fireflies move randomly because they cannot be attracted to any other fireflies. The relationship between fireflies and the objective function value should be established. The absolute brightness of the algorithm is expressed by the objective function value, that is, the absolute brightness *I*_*i*_ of a firefly set at the location $x_{i}^{t}(x_{i1},x_{i2},x_{i3}{,\ldots ,x}_{iD})$ is equal to the objective function value at the location $x_{i}^{t}$, that is

$$I_{i}=f(x_{i}^{t})$$ (1)

Supposing a brighter firefly attracts a firefly with low brightness, the relative brightness between the two fireflies is $I_{ij}(r_{ij})=I_{i}e^{-\gamma r_{ij}^{2}}$. *I*_*i*_ is expressed as the absolute brightness of firefly *i; γ* is the light absorption coefficient, generally set as a constant; *r*_*ij*_ is the cartesian distance from firefly *i* to firefly *j*. That is

$$r_{ij}=\|x_{j}^{t}-x_{i}^{t}\|=\sqrt{\sum_{k=1}^{D}{\left(x_{j}^{t}-x_{i}^{t}\right)}^{2}}$$ (2)

Supposing that the brightness between fireflies is proportional to the attraction, the attraction is *β*_*ij*_,

$$\beta_{ij}(r_{ij})=\beta_{0}e^{-\gamma r_{ij}^{2}}$$ (3)

*β*_0_ is the largest attraction, and generally *β*_0_ = 1.

The updating formula of the algorithm is as follows:

$$x_{i}^{t+1}=x_{i}^{t}+\beta_{ij}(r_{ij})\times (x_{j}^{t}-x_{i}^{t})+\alpha *\delta_{i}$$ (4)

Where, *t* is the number of iterations, *δ*_*i*_ is a random number derived from a uniform distribution, Gaussian distribution, or other distribution, and *α* is a random term coefficient.

**Fig 1:**
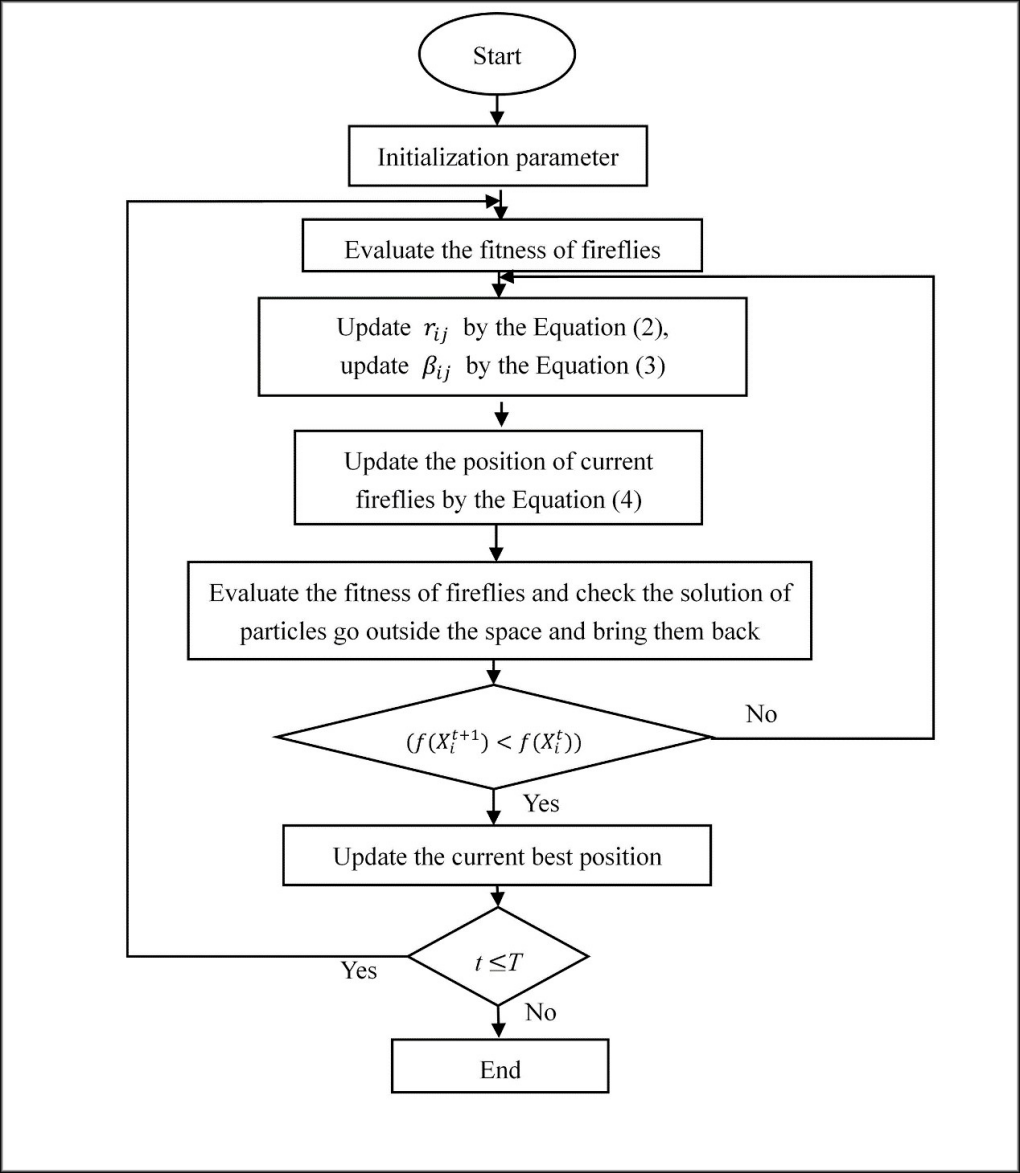
The flow chart of the FA.

The flow chart of the standard firefly algorithm (FA) is shown in the Fig 1:

## 3 The proposed firefly algorithm

Due to the shortcomings of the standard FA, Yang optimized a test function with singularities with FA to achieve better optimization performance. The results show that the FA can effectively solve this kind of global optimization problem, and the FA was successfully applied to the global optimization problem of pressure piping design. However, the parameters in the standard FA are set in advance, which will lead to premature convergence of the algorithm, or the algorithm cannot converge due to improper parameter settings. Hence, the standard FA needs to be improved to achieve better optimization performance.

### 3.1 Parameter analysis of firefly algorithm

The standard FA updates positions based on the attraction between high-brightness fireflies and low-brightness fireflies. Since the visual range of fireflies is limited, low-brightness fireflies can only find mobile higher brightness fireflies within their visual range. It should be noted that the light absorption coefficient *γ* is an important parameter affecting the degree of attraction between fireflies. When *γ*→0, the attraction *β*→*β*_0_. The attraction between fireflies will not change with a change in distance, that is to say, a flash firefly can be seen from anywhere. When *γ*→∞, *β*→0. When the attraction between fireflies is almost zero, individual fireflies move randomly, that is to say, individual fireflies are myopia, which is equivalent to flying in a fog environment; they cannot see each other. The attraction algorithm of fireflies is between these two extremes. When the light absorption coefficient gets a better value (usually 1.0), the distance between two fireflies is the distance that affects the attraction between the two fireflies. If the distance *r*_*ij*_→∞ between two fireflies that are attracted to each other, the attraction *β*→0. Therefore, the basic conditions of the FA in the optimization process are as follows:

1. The distance between attracted low-brightness fireflies and actively high-brightness fireflies should not be too large;
2. There must be a difference in brightness between individual fireflies, with at least one firefly having a higher brightness to attract other fireflies for location updates. There is greater randomness in the distance between individual fireflies that are attracted to each other.

### 3.2 The improvement of firefly algorithm

#### 3.2.1 The minimum attraction

By analysing the parameters of the FA, where the light absorption coefficient γ is too large or where the light absorption coefficient γ is a fixed value, the optimization interval become too large, which will easily result in the attraction between individual fireflies to approach zero and individual fireflies to lose attraction between each other. To avoid the distance between individual fireflies from being too far, the attraction between them may be almost zero. This paper introduces a minimum attraction to improve the standard FA to prevent individual fireflies from randomly moving. At this time,

$$\beta_{ij}(r_{ij})=\beta_{\min}+(\beta_{0}-\beta_{\min})e^{-\gamma r_{ij}^{2}}$$ (5)

*β*_0_ = 1.0, *β*_*min*_∈[0,1]. So, even if the distance between fireflies is too far, $e^{-\gamma r_{ij}^{2}}\to 0$, the attraction between them can be the *β*_*min*_.

**Fig 2:**
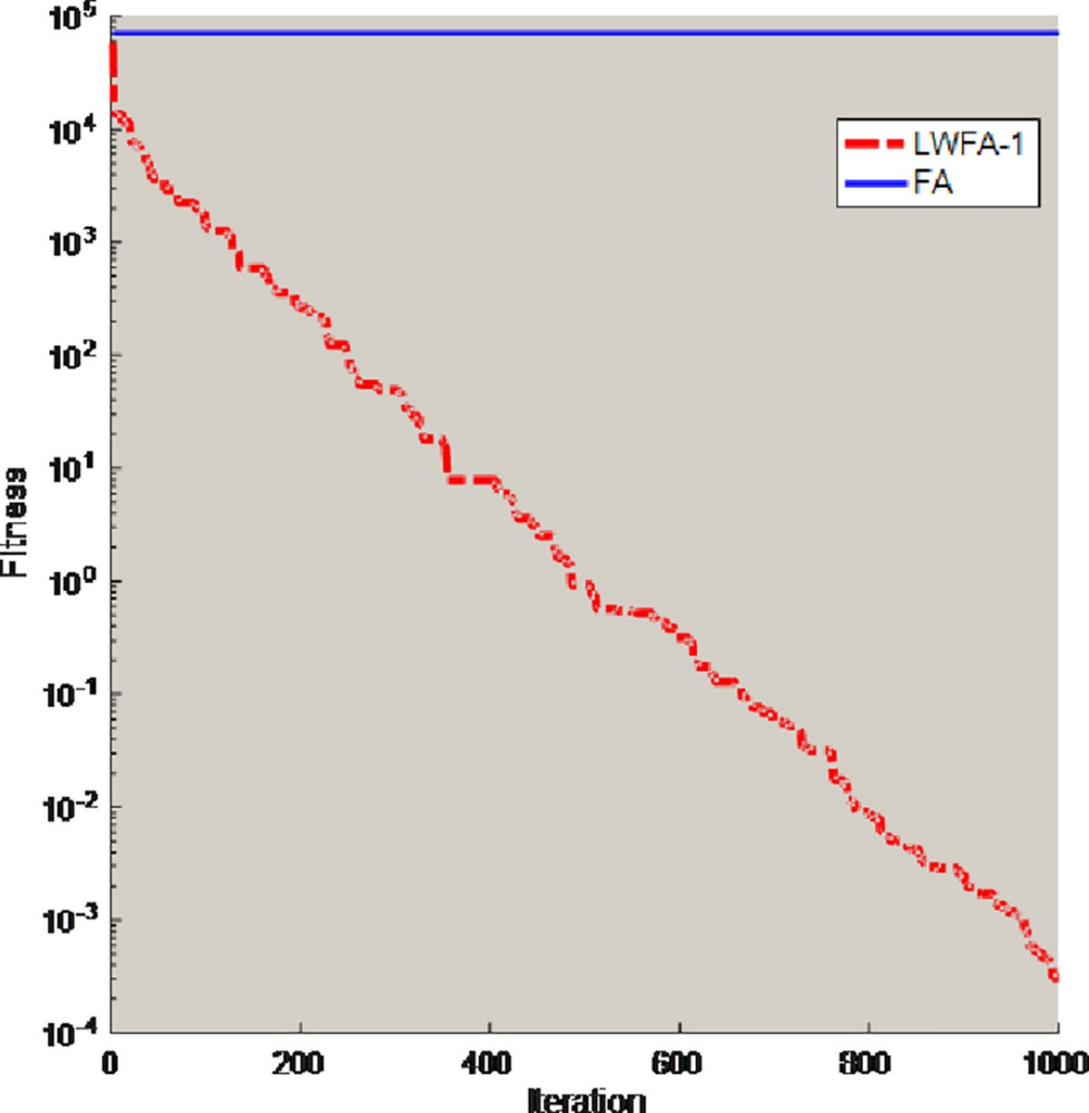
Convergence curves of unimodal functions *f*_2_.

**Fig 3:**
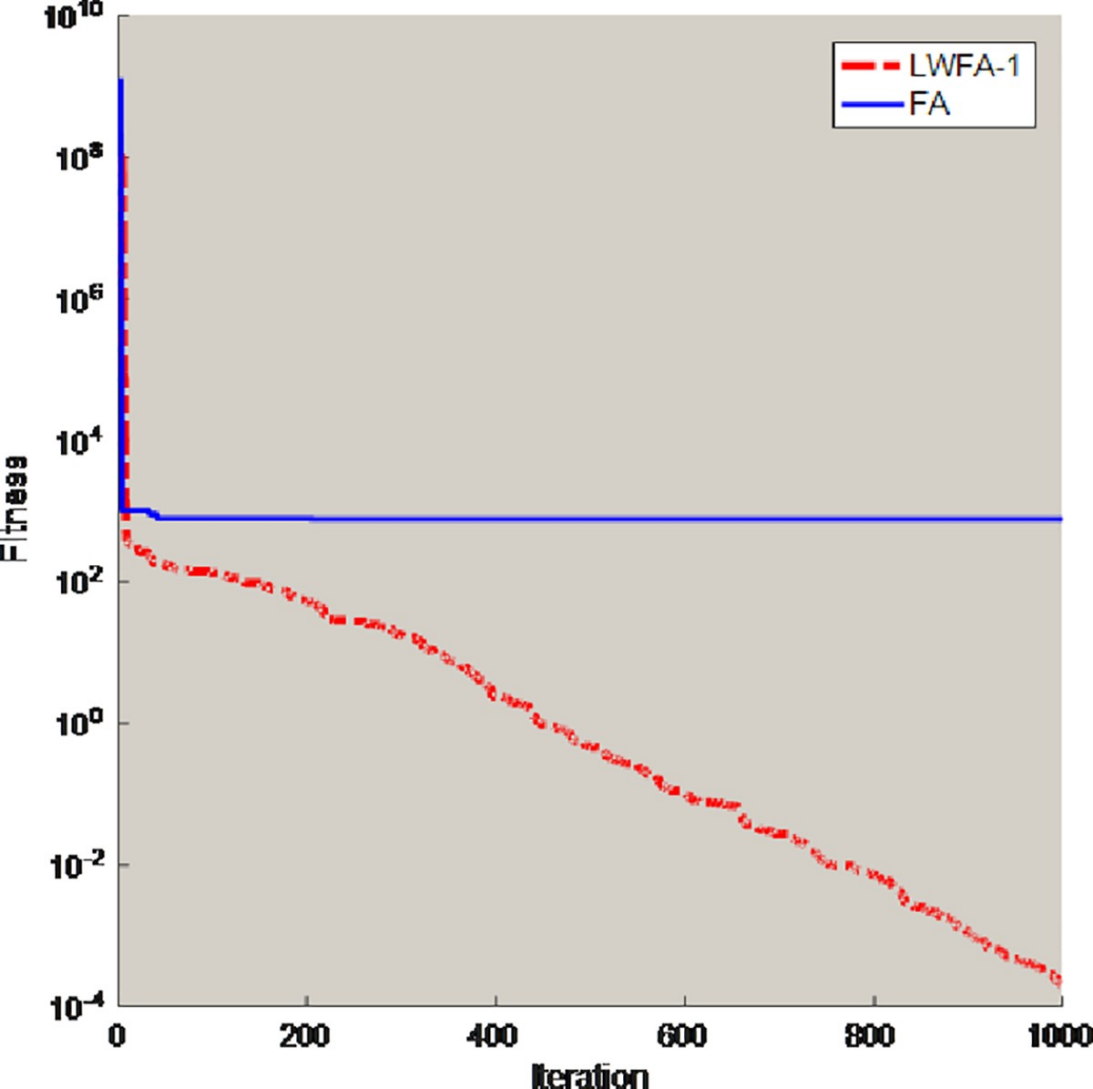
Convergence curves of multimodal functions *f*_7_.

To validate the minimum attraction strategy can solve the shortcomings of the firefly algorithm, some test functions (D = 30) are selected to compare the FA with the algorithm which only obtain the minimum attraction strategy (LWFA-1). The comparison results of best value are shown in Table 1, and this paper also selects two benchmark functions for experiments and draws some conclusions. Fig 2 is the convergence curves of unimodal functions *f*_2_. Fig 3 is the convergence curves of multimodal functions *f*_7_. The population number (N) is 30, and the maximum number of iterations (T) is 1000. All algorithms ran 30 times independently on the test functions.

**Table 1:** The comparison results of LWFA-1 and FA with D = 30.

Function	LWFA-1	FA	Function	LWFA-1	FA
***f***_**1**_	0.1782	0.3122	***f***_**6**_	0.0556	1508.42
***f***_**2**_	3.3580E-04	103.77	***f***_**7**_	9.4238E-05	177.31
***f***_**3**_	60.6925	288.38	***f***_**8**_	0.5163	446.17
***f***_**4**_	0.0075	1.1068	***f***_**9**_	0.0502	6.6831
***f***_**5**_	0.0050	11.782	***f***_**10**_	0.0666	190.87

In the Table 1, the accuracy of the LWFA-1 is greatly reduced. Compared with FA, the LWFA-1 have better performance to solve optimization problems. The comparison results can illustrate the minimum attraction strategy is effect in the LWFA-1. From the Figs 2 and 3, the convergence curve of the FA is basically a straight line and will not change with the increase of iteration times, which indicates that the FA cannot jump out of the local optimal solution. By contrast, converge curve of LWFA-1 is smoother.

Therefore, this strategy can help the original firefly algorithm jump out of the local optimal solution and improve the convergence accuracy of the FA.

#### 3.2.2 Self-Adaptive inertia weight based on logarithmic decrement

The setting of inertia weight *w* can balance the global search ability and local optimization ability to some extent in the iterative process of FA. Initially, the setting of inertia weight is generally linear inertia weight. Based on the motion rule of a firefly in the FA, when a low-brightness firefly is attracted by a brighter individual firefly, the early motion amplitudes of the individual firefly is larger with strong global searchability so that it can enter the process of local optimization at a faster speed. An increase in iteration time helps to bring a firefly closer to the optimal value, and its moving speed should be decreased to improve its local optimization ability and should converge quickly to avoid oscillation state in the vicinity of the optimal value, thus improving the optimization ability of the FA.

**Fig 4:**
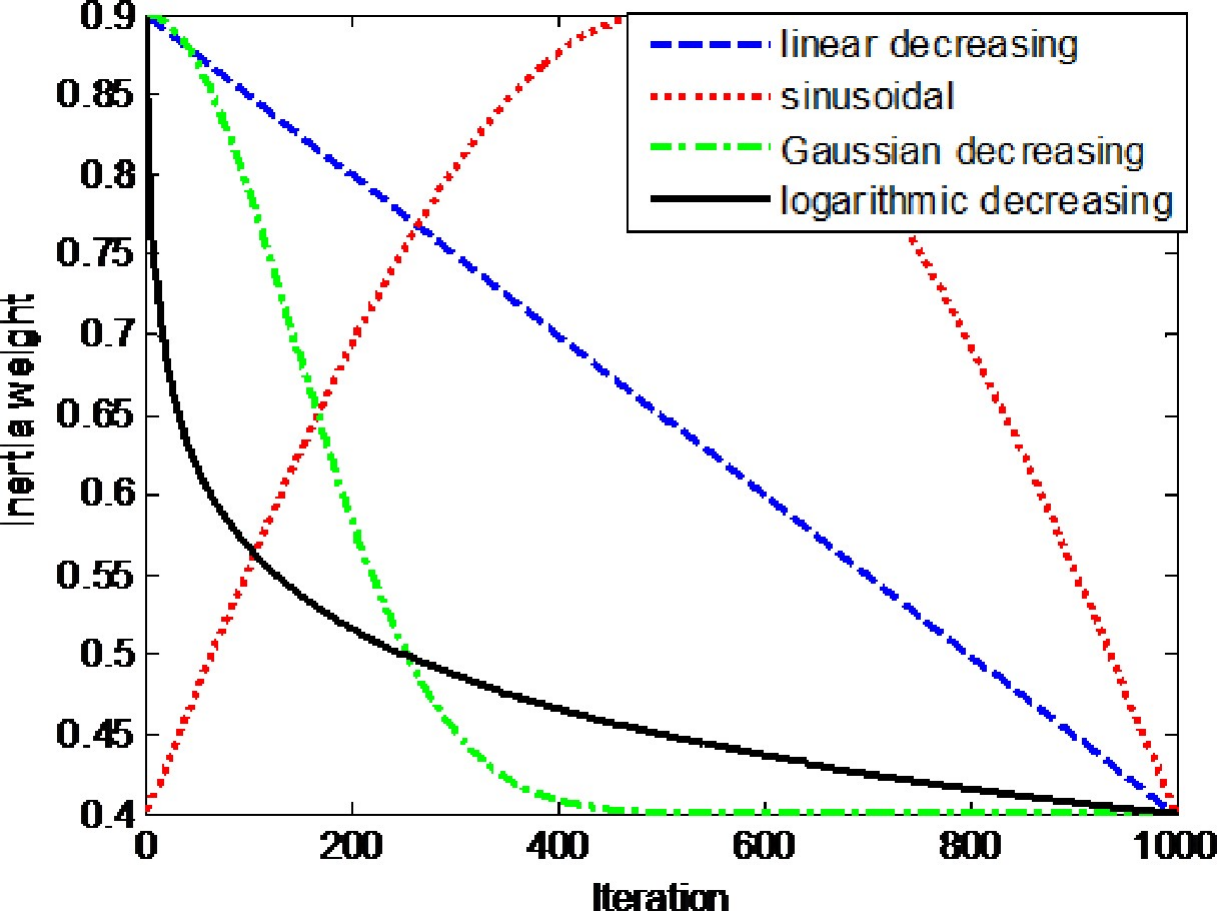
Inertia weight curves for different strategies.

As shown in Fig 4, compared with the linear decreasing inertia weight [29], the inertia weight of sinusoidal adjustment strategy [30], and the Gaussian decreasing inertia weight [31], the logarithmic decreasing self-adaptive inertia weight can be rapidly reduced in the early stages of iteration so that the local search stage can be quickly started and the search speed can be increased. Similarly, compared with the Gaussian decreasing strategy of inertia weight, in the late stages of iteration, the value of inertia weight changes more slowly, and individual fireflies can perform local searches to reduce the occurrence of oscillation. However, Gaussian decreasing inertia weight is almost unchanged after 500 iterations. If a firefly at this time falls into local optimum, it cannot easily jump out of the local optimum position. Therefore, the logarithmic self-adaptive inertia weight strategy is more suitable for individual firefly movement in the FA.

In this paper, logarithmic decreasing self-adaptive inertia weight [32] is used to meet the above requirements. The formula of logarithmic decreasing self-adaptive inertia weight is as follows:

$$w_{t}=w_{1}-b\times (w_{1}-w_{2})\times \log_{T}t$$ (6)

*b* is a logarithmic adjustment factor, *w*_1_ is an initial value of inertia weight, *w*_2_ is a final value of inertia weight, *t* is the current number of iterations, and *T* is the highest number of iterations.

The variation range of the inertial weight coefficient may have an impact on the result of function optimization. In this paper, by reading literature and simulation experiments, the parameter is set to *b* = 1.0、 *w*_1_ = 0.9、 *w*_2_ = 0.4. In this case, the optimize of the firefly algorithm is better.

**Fig 5:**
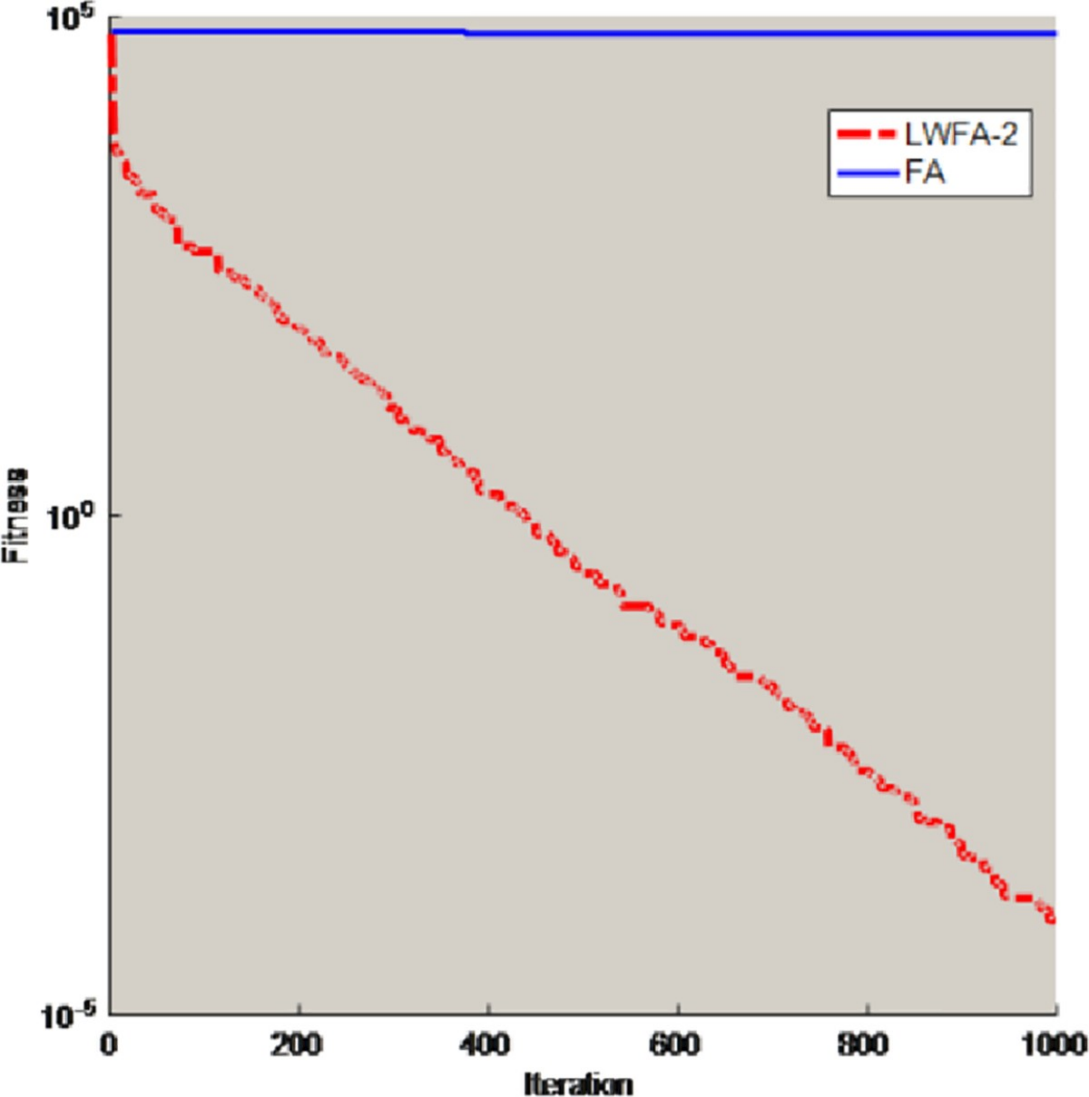
Convergence curves of unimodal functions *f*_2_.

**Fig 6:**
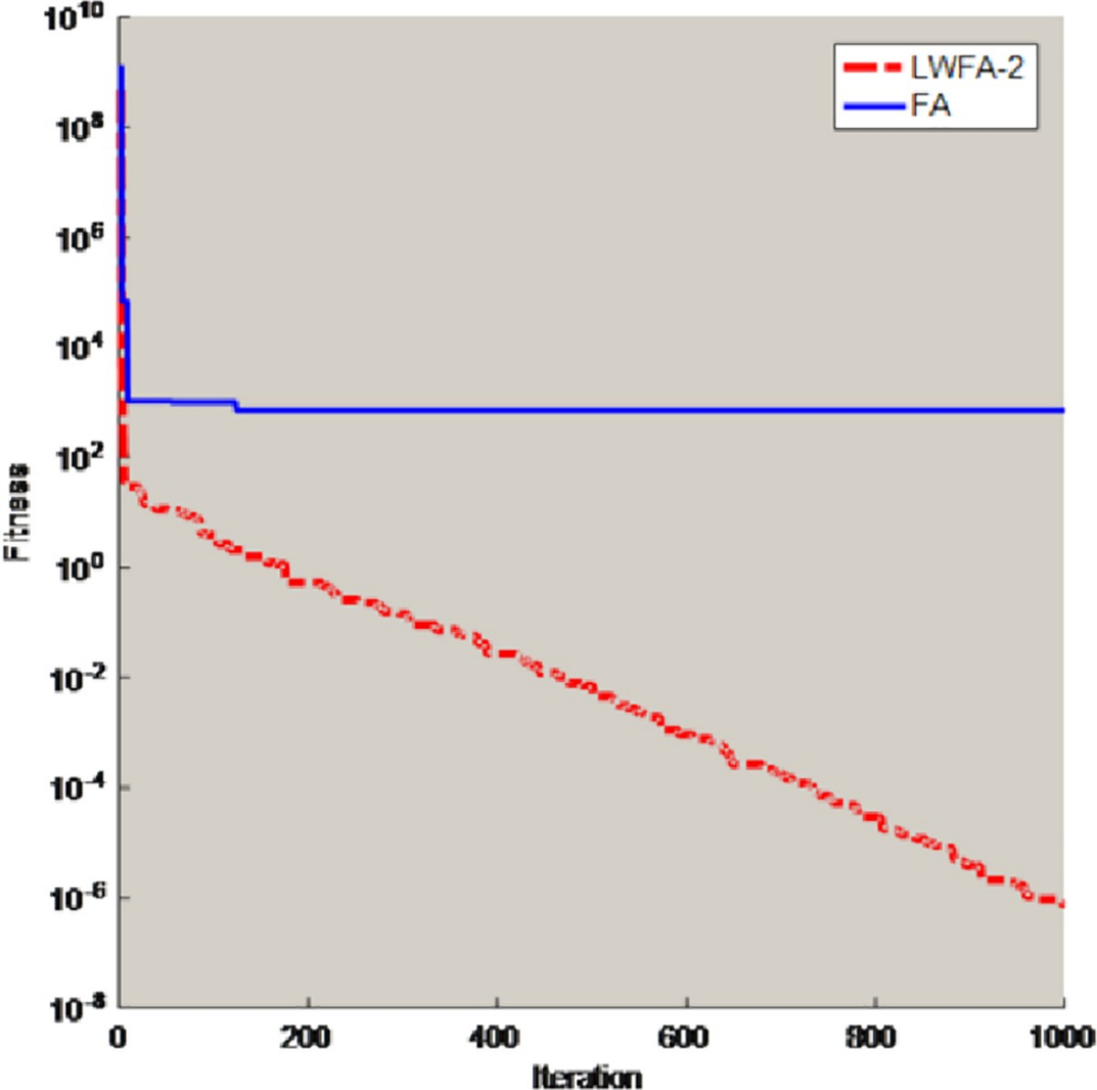
Convergence curves of multimodal functions *f*_7_.

To prove the inertial weight can also solve the shortcomings of the firefly algorithm, some test functions (D = 30) are selected to compare the FA with the algorithm which only obtain the inertial weight (LWFA-2). The comparison results of best value are shown in Table 2, and this paper also selects two benchmark functions for experiments and draws some conclusions. Fig 5 is the convergence curves of unimodal functions *f*_2_. Fig 6 is the convergence curves of multimodal functions *f*_7_. The parameter Settings are the same as in Section 3.2.1.

**Table 2:** The comparison results of LWFA-2 and FA with D = 30.

Function	LWFA-2	FA	Function	LWFA-2	FA
***f***_**1**_	7.0072E-07	0.3122	***f***_**6**_	1.1595E-05	1508.42
***f***_**2**_	7.5761E-05	103.77	***f***_**7**_	5.3248E-07	177.31
***f***_**3**_	5.0291E-05	288.38	***f***_**8**_	1.3483E-06	446.17
***f***_**4**_	3.7239E-06	1.1068	***f***_**9**_	0.0035	6.6831
***f***_**5**_	0.0022	11.782	***f***_**10**_	0.0043	190.87

In the Table 2, compared with FA, the LWFA-2 have better performance to solve optimization problems. From the Figs 5 and 6, the converge curve of LWFA-2 is smoother. Therefore, this strategy also can help the original firefly algorithm jump out of the local optimal solution and improve the convergence accuracy of the FA.

#### 3.2.3 Self-adaptive and dynamic step-size

The random step in the updating formula of the standard FA is generally a random number vector of Gaussian distribution, uniform distribution, or other distribution. Yang introduced a Levy flight into the random part of the updating formula of the FA, which to a certain extent reduces the possibility of individual fireflies falling into local optimal and improves the search performance of the standard FA. At the same time, an increase in the search dimension of an individual firefly decreases the optimization accuracy of the standard FA. For high-dimensional test functions, random turbulence will easily occur and the convergence speed will decrease, and the algorithm cannot converge to an optimal value. Inspired by references [17, 33], this paper introduces the step adjustment factor c.


$$c=\theta^{D}*T*e^{\left(-t/T\right)}$$ (7)

**Fig 7:**
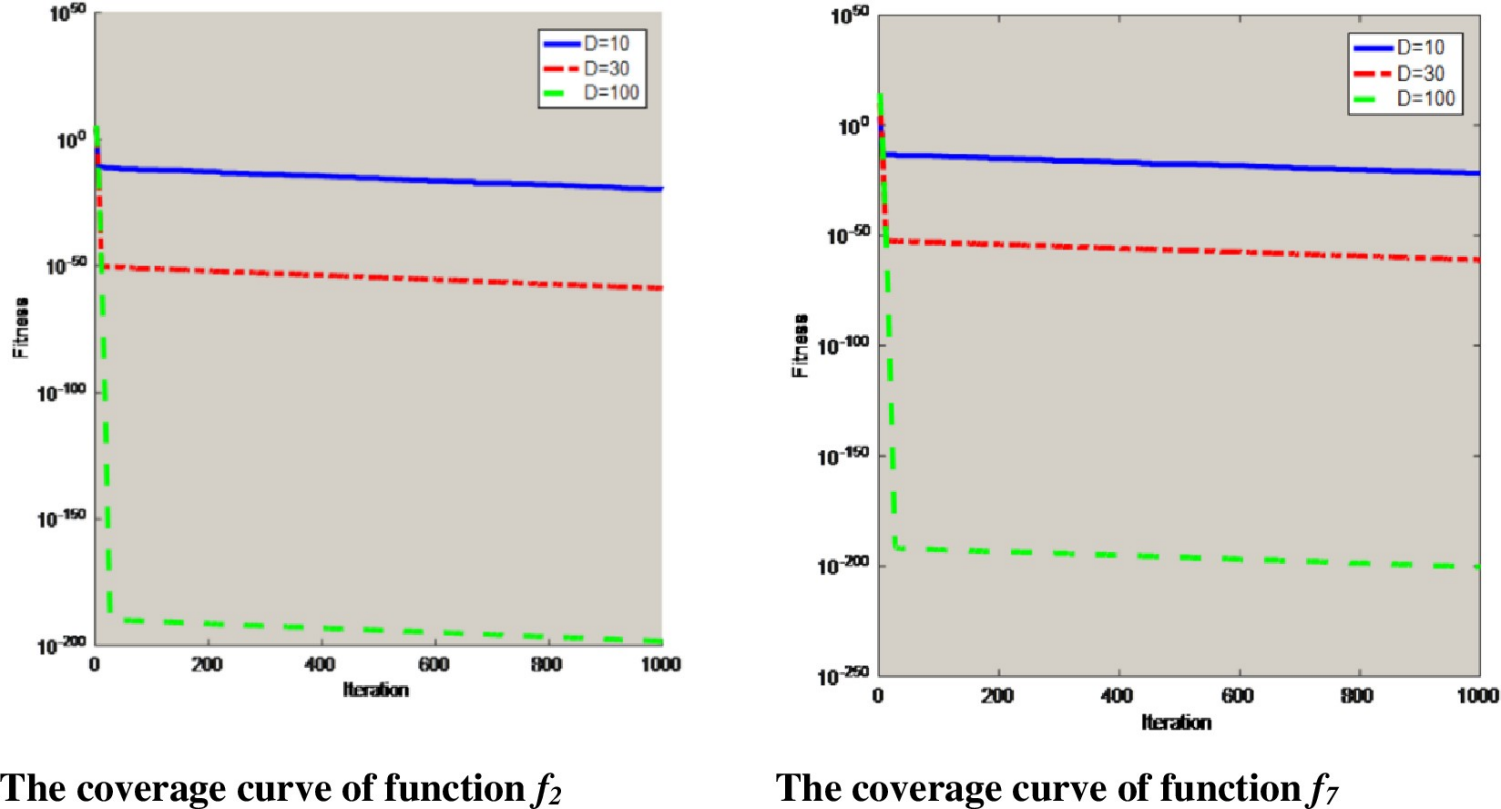
The convergence curves of the function in different dimensions.

*T* is the largest iteration, *t* is the current iteration, *D* is the dimension of an individual firefly, and *θ*∈[0,1]. In this paper, *θ* = 0.1. In the operation of the algorithm, the random step size decreases with an increase in the search dimension of an individual firefly or iteration number. This can ensure that the individual firefly’s random step size change is small in a high-dimensional environment, and accurate explosion can be in a small range to find the optimal value with high precision. To prove that the dynamic step-size strategy can improve the effectiveness of the FA in different dimensions, three dimensions (D = 10/30/100) are used for comparative experiments. This paper selects multimodal function *f*_*2*_ and unimodal function *f*_*7*_ for simulation test on the three dimensions; the population number (N) is 30, and the maximum number of iterations (T) is 1000. Experimental results show that the step adjustment factor can greatly improve the optimization accuracy of the FA. The experimental results are shown in Fig 7.

### 3.3 The procedures for the realization of LWFA

1. In the initial stage, set the following parameters *γ*、 *β*_0_、 *β*_*min*_、 and *α*, firefly population number *N*, and maximum iteration number *T*, and randomly generate the initial position $x_{i}^{t}(i=1,2,\ldots ,n)$ of fireflies in the optimization interval;
2. Substitute the position vector $x_{i}^{t}(i=1,2,\ldots ,n)$ of fireflies into the objective function *f*(*x*) to obtain the initialization brightness *I*_*i*_ of fireflies, and compare and analyze results to obtain the current global optimal brightness *I*_*best*_ and individual optimal positions.
3. Update the phase of the fireflies’ locations. The attraction between individual fireflies *β*_*ij*_.
4. Update the formula according to the position of fireflies. A firefly with high initialization brightness attracts a firefly with low initialization brightness to finalize position updating. The updated formula after improvement is as follows:
$$x_{i}^{t+1}={w_{t}*x}_{i}^{t}+\beta_{ij}(r_{ij})\times (x_{j}^{t}-x_{i}^{t})+\alpha *\delta_{ij}$$ (8)
$$\begin{array}{c} \delta_{ij}=c*rand(1,d)\\ c=\theta^{D}*T*e^{-t/T} \end{array}$$ (9)
5. After updating its position, a firefly generates a new position vector that replaces the old position vector in an objective function, completes the brightness update, and reorders its current brightness to obtain the current global optimal value.
6. Update *w*_*t*_、 *c*、 and *t*. Generally, the stop condition for the iteration in the FA is that the number of iterations reaches a predefined number or that the global optimal result is sought to achieve the required accuracy.

**Fig 8:**
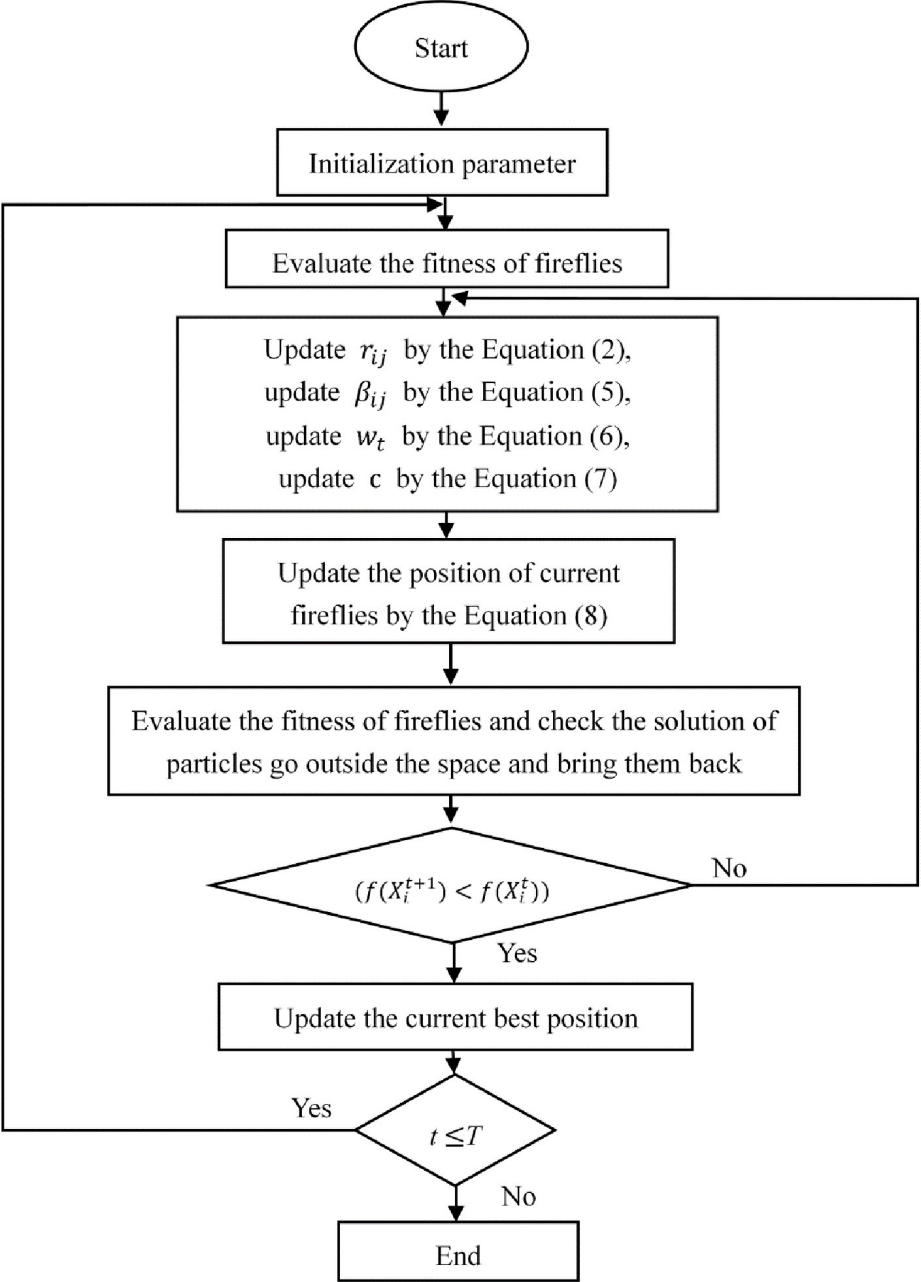
The flow chart of the LWFA.

The flow chart of the LWFA is shown in the Fig 8:

## 4 Theoretical analysis of improved firefly algorithm

### 4.1 The complexity analysis

The complexity of a swarm intelligence algorithm reflects the performance of the algorithm from other different aspects. If the algorithm needs an infinite amount of time to find a global optimal solution, the uptime availability is not included. Therefore, the computational complexity of the algorithm is very significant and crucial. Suppose the population size of the SCA is *N*, the maximum number of iterations is *T*, and the dimension of the decision variable is *D*.

In the standard FA, the computational complexity of the initialization phase is *O (N)*, the positions update of fireflies is O (T*N*D), and checking for fireflies positions outside the search space is O (T*N). Hence, the computational complexity of the FA for the global search for an optimal position is O (N) + O (T*N*D) + O (T*N).

Similarly, the computation complexity of the improved FA in the initialization phase is *O(N)*. The improvement of the minimum attraction is O(T*N), inertia weight based on logarithmic decrement *w*(*t*)_*i*_ is O(T*N), and self-adaptive and dynamic step-size is O(T*N*D). The particle positions update is also O(T*N*D), and checking for particle positions outside the search space is O (T*N). Therefore, the computational complexity of the LWFA to achieve an optimal particle position is O (N) + O (3*T*N) + O (2*T*N*D).

Although the computation complexity of the improved FA (LWFA) is higher than that of the standard FA, both of them are in the same order of magnitude.

### 4.2 The convergence analysis

Convergence analysis is a key factor to evaluate the convergence of swarm intelligence algorithms. As the number of iterations increases, errors between optimal results and theoretical optimal values become minute; eventually, swarm intelligence algorithms approach a fixed value. In the FA, the position update phase of fireflies determines whether the algorithm can converge to a global optimal. Many scholars have used different methods to analyze the convergence of swarm intelligence algorithms. In this paper, the LWFA is analyzed using a second-order nonhomogeneous differential equation.

Using the updating mechanism of the LWFA, we can easily find that the position update of the FA is conducted on a dimension-by-dimensional basis, and each dimension is independent. For the convenience of analysis, this paper simplifies the algorithm by choosing a one-dimensional analysis. From the updating formula (8), weight *w* is the constant coefficient *a*, *β*_*ij*_ is the constant coefficient *b*, and α*δ_*ij*_ is the constant coefficient *d;* x_*j*_(*t*) is the current optimal position of a firefly, denoted as g_*b*_, and the optimal location of the next iteration is denoted as p_*b*_. Thus, the updating formula (8) is reduced as follows:

$$x(t+1)=ax(t)+b\left(g_{b}-x(t)\right)+d$$ (10)

$$x(t+2)=ax(t+1)+b\left(p_{b}-x(t+1)\right)+d$$ (11)

Formulations (10–11) can be obtained as:

$$x(t+2)+(1+r)x(t+1)+rx(t)=bg_{b}+bp_{b}+2d$$ (12)

r=b−a (13)

Its characteristic equation is λ^2^+(1+r)λ+r =0, Δ = (1+*r*)^2^−4*r* = (*r*−1)^2^≥0.

Therefore, the convergence process of LWFA needs to consider the following two conditions:

1) When Δ = 0, the characteristic equation has two same real roots, $\lambda_{1}=\lambda_{2}=\frac{-(1+r)}{2}=-1$, and *x*(*t*) = (*A*_0_+*A*_1_*t*)*λ*^*t*^, *A*_0_, *A*_1_ are undetermined coefficients, the result is calculated as:

$$\left\{ \begin{array}{c} A_{0}=x(0)\\ A_{1}=(r-1)x(0)-bg_{a}-d \end{array}\right.$$

2) When Δ>0, the characteristic equation has two different real roots, $\lambda_{1}=\lambda_{2}=\frac{-(1+r)\pm \sqrt{\Delta }}{2}$, and $x(t)=A_{0}+A_{1}\lambda_{1}^{t}+A_{2}\lambda_{2}^{t}$; *A*_0_, *A*_1_, and *A*_2_ are undetermined coefficients, and the result is calculated as follows:

$$\left\{ \begin{array}{c} A_{0}=x(0)-A_{1}-A_{2}\\ A_{1}=\frac{\lambda_{2}x(0)-(1+\lambda_{2})x(1)+x(2)}{\left(\lambda_{2}-\lambda_{1})(1-\lambda_{1}\right)}\\ A_{2}=\frac{\lambda_{1}x(0)-(1+\lambda_{1})x(1)+x(2)}{\left(\lambda_{2}-\lambda_{1})(1-\lambda_{2}\right)} \end{array}\right.$$

Based on the convergence analysis, if the LWFA converges iteratively, the following two conditions must be met [34].

➀ If t→∞, *x*(0) has its maximum and tend to finite value.
➁ ‖*λ*_1_‖<1, *and* ‖*λ*_2_‖<1

The calculated results are as follows:

When Δ = 0, the convergent domain is r = 1;

When Δ>0, the convergent domain is $\left|\frac{–(1+r)\pm \sqrt{\Delta }}{2}\right|<1\Rightarrow -1<b-a\leq 1$, Thus, $\left\{ \begin{array}{c} b-a\leq 1\\ b-a>-1 \end{array}\right.\Rightarrow \left\{ \begin{array}{c} a\geq b-1\\ a<1+b \end{array}\right.$. According to Eq (5), (6) and (8), the b∈(0,1), *a*∈(−1,2)⇒*w*(*t*)∈(−1,2).

In the whole iteration process of the improved algorithm, the inertia weight region is between 0.4 and 0.9. Therefore, the range of inertia weight conforms to the convergent domain (−1,2), thus the LWFA can converge to a global optimal solution in the iteration.

## 5 Application of improved firefly algorithm in function optimization

To evaluate whether the improved FA has advantages in various applications, two sets of benchmark test functions, ten classical benchmark functions with different dimensions (*D = 10*, *30*, *and 100*) and standard IEEE CEC2010 functions have been used. In this paper, PSO, CS SCA, FPA, FA, the other improved FA and the LWFA are respectively used to conduct simulation experiments, and the experimental results are compared. Based on the experimental results, the improved FA in this paper shows good optimization performance in terms of optimization accuracy and convergence speed.

### 5.1 The benchmark test functions set 1

In this section, ten classical benchmark functions are used as test functions to evaluate the performance of the improved FA algorithm [35]. These functions represent different optimization problems. The specific test functions are as follows:

1. Schaffer function:
$$f_{1}(x)=0.5+\frac{{sin}^{2}\sqrt{\sum_{i=1}^{D}{x_{i}}^{2}}-0.5}{{\left[1+0.001\times \sum_{i=1}^{D}{x_{i}}^{2}\right]}^{2}},x\in [-10,10]$$ (14)
  This function’s theoretical optimal value is 0, and the optimal position is at (0,…,0). This function is continuous, differentiable, non-separable, non-scalable, and unimodal.
2. Sphere function:
$$f_{2}(x)=\sum_{i=1}^{D}x_{i}^{2},x\in [-100,100]$$ (15)
  This function’s minimum value is 0. This function is a classical high-dimensional unimodal function. Its optimal position point (0,…,0) is in the center of the brim, and the relative exploration area is very limited.
3. Rastrigin function:
$$f_{3}(x)=\sum_{i=1}^{D}\left[x_{i}^{2}-10\;\cos(2\pi x_{i})+10\right],x\in [-5.12,5.12]$$ (16)
  This function obtains its optimum value 0 at (0,…,0). This function is a multimodal function, which has ten minimum values in solutions. The peak shape of the function fluctuates in volatility. It is rather difficult to explore global search.
4. Griewank function:
$$f_{4}(x)=\frac{1}{4000}\sum_{i=1}^{D}x_{i}^{2}-\prod_{i=1}^{D}\cos\left(\frac{x_{i}}{\sqrt{i}}\right)+1,x\in [-100,100]$$ (17)
  This function obtains its minimum value 0 at (0,…,0). This function is continuous, differentiable, non-separable, scalable, and multimodal.
5. Ackley function:
$$\begin{array}{c} f_{5}(x)=-20exp\left(-0.2\sqrt{\frac{1}{D}\sum_{i=1}^{D}x_{i}^{2}}\right)-exp\left(\frac{1}{D}\sqrt{\sum_{i=1}^{D}cos(2\pi x_{i}}\right)+20+e,\\ x\in [-35,35] \end{array}$$ (18)
  This function obtains its minimum value 0 at (0,…,0). This function is continuous, differentiable, non-separable, scalable, and multimodal. It has several local optimal values in its search space.
6. Sum-Squares function:
$$f_{6}(x)=\sum_{i=1}^{D}ix_{i}^{2},x\in [-10,10]$$ (19)
  This function’s theoretical optimal value is 0, and its optimal position point is at (0,…,0). This function is continuous, differentiable, separable, scalable, and unimodal.
7. Zakharov function:
$$\begin{array}{c} f_{7}(x)=\sum_{i=1}^{D}x_{i}^{2}+{\left(\frac{1}{2}\sum_{i=1}^{D}ix_{i}\right)}^{2}+{\left(\frac{1}{2}\sum_{i=1}^{D}ix_{i}\right)}^{4},\\ x\in [-5,10] \end{array}$$ (20)
  This function obtains its minimum value 0 at (0,…,0). This function is continuous, differentiable, non-separable, scalable, and multimodal.
8. Schwefel’s problem 1.2 function:
$$f_{8}(x)=\sum_{i=1}^{D}{\left(\sum_{j=1}^{i}x_{j}\right)}^{2},x\in [-10,10]$$ (21)
  This function obtains its minimum value 0 at (0,…,0). This function is continuous, differentiable, non-separable, scalable, and unimodal.
9. Schwefel’s problem 2.21 function:
$$f_{9}(x)=max_{i}\{|x_{i}|,1\leq i\leq D\},x\in [-100,100]$$ (22)
  This function gets the minimum value 0 at (0,…,0). This function is continuous, non-differentiable, separable, scalable, and unimodal.
10. Schwefel’s problem 2.22 function:
$$f_{10}(x)=\sum_{i=1}^{D}\left|x_{i}\right|+\prod_{i=1}^{D}\left|x_{i}\right|,x\in [-10,10]$$ (23)
  This function obtains its minimum value 0 at (0,…,0). This function is continuous, differentiable, non-Separable, scalable, and unimodal.

### 5.2 Comparison with other improved firefly algorithms on set 1

In this section, some improved firefly algorithms are selected to prove the optimization ability of LWFA. The results of other improved FA are extracted from the original literatures. The comparison results of LWFA, VVSFA [36], IWFA [37], and CLFA [38] are shown in the Table 3.

**Table 3:** Comparison results of LWFA and other improved FA with D = 100.

Function	VVSFA	IWFA	CLFA	LWFA
***f***_***2***_	84.843	1.0028	7.5021	5.42E-201
***f***_***3***_	470.286	177.324	561.675	0
***f***_***4***_	0.74506	0.02078	0.16899	0
***f***_***5***_	4.84312	0.84394	3.60037	0
***f***_***6***_	3853.70	51.513	364.380	2.04E-199
***f***_***8***_	3828.88	50.2529	374.359	2.02E-101
***f***_***10***_	64.584	1.64239	9.0614	5.81E-100

From the Table 3, the average value is contained. In the same dimension D = 100, the accuracy of LWFA is obviously higher than other improved algorithms. When solve the high dimension optimization problems, compared with other FA, the LWFA can overcome the curse of dimensionality. Therefore, the LWFA is competitive in solving optimization problems.

### 5.3 Comparison with other state-of-art algorithms on set 1

For the ten test functions, *f*_*2*_, *f*_*6*_, *f*_*8*_, *f*_*9*_, and *f*_*10*_ are unimodal functions, and *f*_*1*_, *f*_*3*_, *f*_*4*_, *f*_*5*_, and *f*_*7*_ are multimodal functions. The dimensions of the test functions are set to 10, 30, and 100, respectively. The test results are obtained using PSO, CS algorithm, SCA, FPA, FA, and LWFA.

The experimental environment is MATLAB 2014a, the operating system is windows 7 flagship, 4.00 GB of running memory and the processor is Intel (R) Core (TM) i3 – 2350M CPU @ 2.30 GHz. The parameters of the six algorithms PSO [33], the CS [34], the FPA [35], the SCA [8], the FA [36], and the LWFA are as shown in Table 4.

**Table 4:** The parameters of POS, CS, SCA, FPA, FA, LWFA.

Algorithm	Parameters values
**PSO**	*c*_1_ = *c*_2_ = 1.494, *w*∈[0.4,0.9], *V*_*max*_ = 1, *V*_*min*_ = −1, *N* = 30, *T* = 1000
**FPA**	*P =* 0.2, *N = 30*, *T =* 1000
**SCA**	*a* = 2, *N* = 30, *T* = 1000
**CS**	*P*_*A*_ = 0.25, *N* = 30, *T* = 1000
**FA**	α = 1.0, *β*_0_ = 1.0, *γ* = 1.0, *N* = 30, *T* = 1000
**LWFA**	α = 1.0, *β*_0_ = 1.0, *β*_*min*_ = 0.2, *γ* = 1.0, *N* = 30, *T* = 1000

To ensure fairness and comparability in the simulation experiments, the same experimental parameters are defined for all the algorithms. Each algorithm will run 30 times to solve each test function, and the test results will be recorded; the optimal solution of the objective function value, the worst solution, the average value, and standard deviation are obtained in this dimension, as shown in Tables 5–7.

**Table 5:** The optimization result of 10 benchmark functions for different algorithms (D = 10).

function	algorithm	the best value	the worst value	the average value	standard deviation	function	algorithm	the best value	the worst value	the average value	standard deviation
***f***_***1***_	PSO	0.0097	0.0782	0.0440	0.0209	***f***_***6***_	PSO	1.6059E-13	9.4905E-05	8.1838E-06	2.1168E-05
	CS	0.0097	0.0372	0.0116	0.0070		CS	5.2625E-15	2.8228E-12	3.2843E-13	6.3288E-13
	FPA	0.0099	0.0429	0.0357	0.0071		FPA	7.5162E-04	0.0221	0.0047	0.0043
	SCA	0.0097	0.0097	0.0097	2.3360E-07		**SCA**	**1.8623E-35**	**1.2869E-25**	**6.3602E-27**	**2.5140E-26**
	FA	4.3300E-02	1.2710E-01	9.4133E-02	2.2880E-02		FA	3.1187E+01	7.8010E+01	4.8660E+01	1.2126E+01
	**LWFA**	**0**	**5.5511E-17**	**3.8858E-17**	**2.5873E-17**		LWFA	3.0366E-22	1.0505E-21	6.7096E-22	1.8083E-22
***f***_***2***_	PSO	1.1811E-11	4.5714E-06	3.3055E-07	8.9581E-07	***f***_***7***_	PSO	1.6577E-06	26.0726	0.8697	4.7601
	CS	1.8841E-15	9.1193E-14	1.9124E-14	1.8349E-14		CS	3.6268E-07	2.3082E-04	3.0668E-05	4.8888E-05
	FPA	0.0200	0.2283	0.0853	0.0514		FPA	3.0419E-04	0.0034	0.0017	7.9478E-04
	**SCA**	**7.1155E-37**	**6.4354E-25**	**2.8900E-26**	**1.1783E-25**		SCA	2.0934E-21	2.4024E-11	8.0332E-13	4.3856E-12
	FA	4.7507E+00	1.5125E+01	1.0938E+01	2.2635E+00		FA	9.9440E+00	3.8105E+01	2.5361E+01	6.9977E+00
	LWFA	1.6529E-23	5.8901E-23	3.7995E-23	1.0073E-23		**LWFA**	**3.3720E-23**	**1.0120E-22**	**5.8941E-23**	**1.7883E-23**
***f***_***3***_	PSO	3.9832	26.9286	10.7593	5.0800	***f***_***8***_	PSO	3.0130E-07	0.0257	0.0013	0.0046
	CS	4.2552	10.0941	6.5688	1.5021		CS	5.6553E-10	3.3123E-08	7.5349E-09	6.9291E-09
	FPA	10.4006	26.9615	18.6496	4.2546		FPA	1.3139E-04	0.0014	5.8059E-04	2.9570E-04
	SCA	0	1.5447E-06	5.6132E-08	2.8229E-07		SCA	1.6318E-19	8.4752E-10	3.0634E-11	1.5440E-10
	FA	4.7599E+01	8.1388E+01	6.4081E+01	8.4711E+00		FA	2.7464E+01	6.3785E+01	4.6410E+01	9.8794E+00
	**LWFA**	**0**	**0**	**0**	**0**		**LWFA**	**4.7114E-23**	**2.2662E-22**	**1.6560E-22**	**4.7540E-23**
***f***_***4***_	PSO	0.0640	0.7308	0.2980	0.1454	***f***_***9***_	PSO	0.0023	0.0865	0.0411	0.0256
	CS	0.0149	0.0850	0.0454	0.0161		CS	1.7888E-04	7.1200E-04	3.7998E-04	1.4049E-04
	FPA	0.1440	0.4735	0.3126	0.0680		FPA	0.4786	1.9163	1.0946	0.3118
	SCA	0	0.5153	0.0390	0.1128		SCA	7.7359E-12	8.4083E-07	6.7627E-08	1.7704E-07
	FA	3.0980E-01	6.4260E-01	4.9305E-01	7.7488E-02		FA	2.0423E+00	4.2122E+00	3.3785E+00	5.5880E-01
	**LWFA**	**0**	**0**	**0**	**0**		**LWFA**	**4.8645E-12**	**8.2962E-12**	**6.5972E-12**	**8.3867E-13**
***f***_***5***_	PSO	4.4785E-07	1.1581	0.1937	0.4377	***f***_***10***_	PSO	0.0015	0.1362	0.0290	0.0323
	CS	2.1457E-05	0.0776	0.0058	0.0152		CS	5.8613E-07	9.2833E-06	3.2644E-06	2.2526E-06
	FPA	0.4717	3.1001	1.3024	0.5851		FPA	0.1960	1.3147	0.4822	0.2314
	**SCA**	**0**	**9.2371E-14**	**1.6579E-14**	**2.4584E-14**		SCA	1.4191E-23	4.7817E-18	2.9770E-19	9.2584E-19
	FA	5.8071E+00	9.4430E+00	8.4697E+00	8.1240E-01		FA	9.4411E+00	2.0651E+01	1.6371E+01	2.4332E+00
	LWFA	1.0807E-11	1.8442E-11	1.4506E-11	2.0414E-12		**LWFA**	**2.1315E-11**	**3.4736E-11**	**2.8367E-11**	**2.9999E-12**

**Table 6:** The optimization result of 10 benchmark functions for different algorithms (D = 30).

function	algorithm	the best value	the worst value	the average value	standard deviation	function	algorithm	the best value	the worst value	the average value	standard deviation
***f***_***1***_	PSO	0.0782	0.1782	0.1306	0.0262	***f***_***6***_	PSO	1.7531	25.0448	6.7477	4.5681
	CS	0.0373	0.0782	0.0752	0.0104		CS	8.2527E-05	8.4290E-04	4.0109E-04	1.9621E-04
	FPA	0.0372	0.0782	0.0742	0.0123		FPA	23.6230	154.3394	76.1294	33.2436
	SCA	0.0097	0.0782	0.0308	0.0183		SCA	1.3975E-07	0.0047	5.0452E-04	0.0010
	FA	3.1220E-01	3.4570E-01	3.3796E-01	1.2084E-02		FA	1.5084E+03	1.9225E+03	1.7262E+03	1.2639E+02
	**LWFA**	**0**	**0**	**0**	**0**		**LWFA**	**7.6511E-61**	**1.9997E-60**	**1.5032E-60**	**2.4686E-61**
***f***_***2***_	PSO	0.1902	1.0520	0.4540	0.2149	***f***_***7***_	PSO	15.0176	212.2121	63.6612	49.9723
	CS	8.6604E-04	0.0075	0.0031	0.0017		CS	88.2473	210.1480	129.1037	29.7051
	FPA	262.1592	1.2650E+03	661.7135	226.6214		FPA	30.3362	85.3530	53.1830	15.3680
	SCA	2.0687E-06	0.7171	0.0339	0.1308		SCA	0.4161	15.2616	4.5798	3.6660
	FA	1.0377E+02	1.4254E+02	1.2540E+02	9.3352E+00		FA	1.7731E+02	2.3392E+02	2.0774E+02	1.5899E+01
	**LWFA**	**8.9383E-62**	**1.3154E-61**	**1.1222E-61**	**1.4028E-62**		**LWFA**	**2.9461E-62**	**5.7812E-62**	**4.2535E-62**	**6.2112E-63**
***f***_***3***_	PSO	42.8954	136.2840	92.5186	23.5156	***f***_***8***_	PSO	7.1718	43.5822	19.3772	9.3495
	CS	56.8094	111.4573	81.1353	13.4428		CS	1.4197	4.2732	2.6419	0.7065
	FPA	103.9302	150.2750	125.5139	12.2622		FPA	1.2049	6.6488	3.6693	1.5667
	SCA	1.3988E-04	80.4870	14.6301	19.0388		SCA	1.2531	135.7837	37.6145	29.5912
	FA	2.8838E+02	3.8032E+02	3.5731E+02	1.8252E+01		FA	4.4617E+02	6.8185E+02	5.5592E+02	5.4870E+01
	**LWFA**	**0**	**0**	**0**	**0**		**LWFA**	**1.0874E-61**	**2.9668E-61**	**1.7842E-61**	**3.9272E-62**
***f***_***4***_	PSO	0.0128	0.1849	0.0475	0.0355	***f***_***9***_	PSO	1.4777	18.7225	7.3092	4.3077
	CS	7.5014E-04	0.0536	0.0101	0.0129		CS	2.4960	10.1774	5.6504	1.7214
	FPA	2.6201	9.4595	6.4769	1.8591		FPA	10.3986	18.4567	14.2229	2.1451
	SCA	1.3316E-08	0.9080	0.1774	0.2850		SCA	1.1044	41.0334	20.5064	12.7204
	FA	1.1068E+00	1.1342E+00	1.1219E+00	7.2960E-03		FA	6.6831E+00	8.0281E+00	7.5500E+00	3.4282E-01
	**LWFA**	**0**	**0**	**0**	**0**		**LWFA**	**1.0002E-31**	**1.3789E-31**	**1.2292E-31**	**9.3718E-33**
***f***_***5***_	PSO	0.4425	3.2575	2.3851	0.5495	***f***_***10***_	PSO	0.9885	11.9882	4.0160	3.2955
	CS	56.8094	111.4573	81.1353	13.4428		CS	0.0609	0.2279	0.1333	0.0453
	FPA	3.1234	7.4835	4.8481	1.2431		FPA	10.4606	25.5790	15.8913	3.0222
	SCA	2.8420E-04	20.3911	18.4823	5.5056		SCA	6.2089E-08	4.5451E-04	2.9099E-05	8.4071E-05
	FA	1.1782E+01	1.3155E+01	1.2664E+01	3.1784E-01		FA	1.9087E+02	6.9022E+07	4.1621E+06	1.2682E+07
	**LWFA**	**0**	**0**	**0**	**0**		**LWFA**	**1.1946E-30**	**1.5411E-30**	**1.3823E-30**	**8.5811E-32**

**Table 7:** The optimization result of 10 benchmark functions for different algorithms (D = 100).

function	algorithm	the best value	the worst value	the average value	standard deviation	function	algorithm	the best value	the worst value	the average value	standard deviation
***f***_***1***_	PSO	0.2277	0.3733	0.3066	0.0295	***f***_***6***_	PSO	877.5363	2.0832e+03	1.3193e+03	289.6528
	CS	0.1782	0.2277	0.1931	0.0231		CS	69.3377	205.6963	120.0117	34.0957
	FPA	0.1270	0.2290	0.1768	0.0316		FPA	2.1184E+03	4.4187E+03	3.3137E+03	594.5536
	SCA	0.0814	0.3123	0.2146	0.0463		SCA	229.7247	6.0798E+03	1.6547E+03	1.3282E+03
	FA	4.5990E-01	4.7390E-01	4.6922E-01	3.9257E-03		FA	2.8050E+04	3.4829E+04	3.2328E+04	1.6309E+03
	**LWFA**	**0**	**0**	**0**	**0**		**LWFA**	**2.1345E-199**	**3.0341E-199**	**2.6422E-199**	**0**
***f***_***2***_	PSO	19.9744	79.6507	46.6166	12.1838	***f***_***7***_	PSO	8.1925E+03	2.7431E+04	1.3991E+04	4.6078E+03
	CS	159.7210	456.8990	276.0323	77.7914		CS	870.9981	1.5087E+03	1.2052E+03	166.0680
	FPA	5.7575E+03	9.8960E +03	7.4427E+03	962.7697		FPA	585.9580	1.4666E+03	991.1530	222.2277
	SCA	337.8922	1.9765e+04	5.4671e+03	5.4292E+03		SCA	133.4625	288.3350	195.5669	40.9209
	FA	6.3068E+02	7.0802E+02	6.6869E+02	2.2472E+01		FA	7.8111E+02	1.0451E+03	9.2988E+02	6.8783E+01
	**LWFA**	**4.5054E-201**	**6.0252E-201**	**5.4209E-201**	**0**		**LWFA**	**1.5016E-201**	**2.4386E-201**	**2.0039E-201**	**0**
***f***_***3***_	PSO	490.3816	776.5889	606.5859	59.7708	***f***_***8***_	PSO	339.0808	815.9485	526.2622	116.3743
	CS	334.8207	481.6886	428.7982	39.9027		CS	227.1708	459.1311	321.5536	49.0437
	FPA	589.6294	734.1776	674.4609	36.4772		FPA	40.0958	111.0140	66.1648	16.9574
	SCA	1.2247	558.7510	242.6099	119.8851		SCA	1.2439E+03	2.9323E+03	1.8525E+03	407.4930
	FA	1.4705E+03	1.5829E+03	1.5373E+03	2.5691E+01		FA	4.2350E+03	6.3283E+03	5.4018E+03	5.3043E+02
	**LWFA**	**0**	**0**	**0**	**0**		**LWFA**	**1.1439E-200**	**2.8352E-200**	**2.0264E-200**	**0**
***f***_***4***_	PSO	0.4433	0.6773	0.5852	0.0600	***f***_***9***_	PSO	20.6272	30.6745	25.5670	2.8529
	CS	0.9339	1.1244	1.0449	0.0440		CS	17.3485	28.3434	23.0299	2.8894
	FPA	53.3739	105.0862	68.9915	13.0304		FPA	20.4611	29.4837	24.9385	2.4659
	SCA	0.6948	5.1392	2.2343	1.0284		SCA	72.8254	92.1978	85.7399	4.7084
	FA	1.5571E+00	1.6804E+00	1.6402E+00	2.5173E-02		FA	9.3510E+00	9.7162E+00	9.5422E+00	1.0333E-01
	**LWFA**	**0**	**0**	**0**	**0**		**LWFA**	**1.5683E-101**	**1.8175E-101**	**1.7278E-101**	**5.5112E-103**
***f***_***5***_	PSO	6.1351	14.7410	10.3604	2.7097	***f***_***10***_	PSO	25.2204	62.2287	39.4478	9.2937
	CS	9.1747	19.7776	13.6876	3.3292		CS	10.0777	19.7471	13.3932	2.1460
	FPA	4.5747	7.8755	6.1055	0.9323		FPA	57.6723	83.5676	71.6025	6.1407
	SCA	20.5231	20.6970	20.6298	0.0380		SCA	0.1123	5.1104	1.4292	1.3773
	FA	1.3993E+01	1.4587E+01	1.4347E+01	1.2047E-01		FA	8.9329E+37	1.8601E+43	1.6162E+42	4.2571E+42
	**LWFA**	**0**	**0**	**0**	**0**		**LWFA**	**5.3091E-100**	**6.2809E-100**	**5.8188E-100**	**2.1445E-101**

Based on Tables 5–7, when D = 10/30/100, the LWFA significantly improves convergence accuracy. For either unimodal functions or multimodal functions that have multiple extreme points, as the dimension increases, the LWFA obtains better optimal performance. When D = 100, the accuracy of LWFA is more than 100 magnitudes higher than when D = 10. The LWFA can also solve optimization problems with a small standard deviation, indicating that the improved algorithm has greater robustness to solve high-dimension problems.

By comparing Tables 5–7, we can find that when D = 10, the LWFA can obtain optimal value 0 for functions *f*_*1*_, *f*_*3*,_ and *f*_*4*_, while the optimization accuracy of the other four algorithms (PSO, the CS, FPA, and FA) is not high. For functions *f*_*2*,_ *f*_*5*_*–f*_*10*_, the LWFA proposed in this paper is more accurate than PSO, the CS, and FA in terms of optimal values and standard deviation. The LWFA has higher convergence accuracy than the SCA except for the functions *f*_*2*_, *f*_*5–6*_. When D = 30, the standard CS is used to solve *f*_*6*_ and *f*_*10*_, and it failed many times in the convergence experiments. Moreover, when D = 100, PSO is unable to solve the convergence problem for the function *f*_*7*_. The CS also loses convergence for functions *f*_*3*_, *f*_*6*_, *f*_*8*_, and *f*_*10*._ When D = 100, the LWFA can quickly reach the optimal value 0 for functions *f*_*1*_, *f*_*3*_, *f*_*4*_, and *f*_*5*_. For other functions, the optimization accuracy and stability of PSO, the CS, FPA, and FA is lower than the performance of LWFA proposed in this paper. Particularly, the performance of the SCA decreases rapidly as the dimension increases, but the LWFA has a faster convergence speed and higher accuracy in 30 high-dimensional independent runs (D = 100).

### 5.4 Experiments and results analysis on set 2

In this section, to further analyze the performance of the improved FA, IEEE CEC2010 functions [39] are used in simulation experiments. PSO, the SCA, the FPA, and the standard FA are used to evaluate the performance of the LWFA. The simulation environment is similar to that of set 1. The parameters of the five algorithms are also shown in Table 4. To realize significant solutions, each function is run 30 times independently, and all the simulation experiments are tested under the same conditions. The solutions of all the algorithms are shown in Table 8.

**Table 8:** The result of CEC2010 standard functions for different algorithms (D = 30).

function	Index	PSO	FPA	SCA	FA	LWFA
***f***_***1***_	**Mean**	1.1758E+08	2.8482E+07	3.2023E+08	3.2758E+09	1.2335E+09
	**St.dev**	1.2266E+08	9.8650E+06	8.3177E+07	6.6525E+08	2.9624E+08
***f***_***2***_	**Mean**	194.4268	270.7726	313.9159	429.0046	487.6912
	**St.dev**	22.5446	24.3295	18.1015	22.3716	18.0347
***f***_***3***_	**Mean**	6.9845	11.5825	8.4616	9.9132	20.6573
	**St.dev**	5.0723	0.8924	0.4262	0.1842	0.1956
***f***_***4***_	**Mean**	1.9000E+09	1.1098E+08	1.6428E+09	8.6480E+10	5.2756E+09
	**St.dev**	2.5837E+09	3.4122E+07	6.3352E+08	1.1906E+11	1.4965E+09
***f***_***5***_	**Mean**	843.4820	341.8182	2.1540E+03	1.3410E+04	5.1709E+03
	**St.dev**	159.9266	28.5857	1.3582E+03	9.2452E+04	1.3000E+03
***f***_***6***_	**Mean**	1.6696E+06	29.0407	8.9861E+04	3.2047E+06	5.8606E+05
	**St.dev**	1.9389E+06	7.4758	6.9757E+04	1.1767E+06	4.1916E+05
***f***_***7***_	**Mean**	4.1934E+07	3.5154E+04	2.3353E+05	7.4838E+07	3.3784E+06
	**St.dev**	1.0738E+05	8.0394E+03	7.2418E+04	8.5399E+07	3.5215E+06
***f***_***8***_	**Mean**	4.5953E+13	1.7543E+05	2.5375E+06	5.9478E+11	2.2544E+07
	**St.dev**	1.9726E+12	4.3275E+04	3.2598E+06	7.9379E+11	1.1232E+07
***f***_***9***_	**Mean**	1.3289E+07	1.9022E+07	7.0679E+07	4.4532E+08	7.2144E+08
	**St.dev**	1.8736E+07	7.0583E+06	1.7118E+07	9.4426E+07	1.5339E+08
***f***_***10***_	**Mean**	112.8304	158.8623	179.4504	303.8515	268.3395
	**St.dev**	34.0315	16.8684	19.1803	29.1816	20.2220
***f***_***11***_	**Mean**	40.7407	28.7450	34.2656	64.0681	51.6848
	**St.dev**	5.4358	2.0124	3.8885	4.3817	2.6954
***f***_***12***_	**Mean**	2.7810E+04	1.4545E+04	2.3914E+04	5.5761E+04	8.4959E+04
	**St.dev**	1.8096E+03	3.9676E+03	2.2166E+03	4.5165E+03	8.8299E+03
***f***_***13***_	**Mean**	4.4553E+09	4.1246E+05	4.8125E+06	4.4521E+09	1.3288E+08
	**St.dev**	7.5115E+08	3.1061E+05	1.8795E+06	2.0723E+09	1.2952E+08
***f***_***14***_	**Mean**	4.1494E+07	6.0881E+07	2.5674E+08	1.5656E+09	9.1115E+08
	**St.dev**	5.4354E+07	4.8423E+07	9.2009E+07	2.0959E+08	1.0834E+08
***f***_***15***_	**Mean**	46.9529	92.8979	99.1639	166.1725	173.0482
	**St.dev**	10.6700	9.1652	12.0043	18.0469	11.9303
***f***_***16***_	**Mean**	46.6296	44.6271	46.9118	71.5064	73.7265
	**St.dev**	8.8237	4.1806	3.2170	3.9258	2.6880
***f***_***17***_	**Mean**	5.2609E+04	1.4721E+04	2.9294E+04	1.0348E+05	9.4555E+04
	**St.dev**	1.4283E+04	4.0175E+03	5.4200E+03	1.0450E+04	1.2981E+04
***f***_***18***_	**Mean**	1.0972E+10	N/A	3.5760E+08	2.5780E+10	7.0092E+09
	**St.dev**	2.8496E+09	N/A	1.5551E+08	6.6432E+09	2.2750E+09
***f***_***19***_	**Mean**	3.1396E+04	1.6728E+04	3.4017E+04	1.1001E+05	1.1440E+05
	**St.dev**	7.3101E+03	5.7618E+03	6.6369E+03	2.1627E+04	1.9785E+04
***f***_***20***_	**Mean**	7.1139E+09	2.4342E+09	2.3806E+09	2.2325E+10	4.6233E+10
	**St.dev**	8.4790E+08	1.0194E+09	7.0705E+08	4.1391E+09	7.4252E+09

The details of the results—mean and standard deviation are recorded in Table 6. Based on Table 8, it can be easily analyzed that the LWFA outperformed the standard FA. Compared with the other algorithms (PSO, the FPA, FA, and SCA), the im-proved FA achieved superior optimization performance.

### 5.5 Convergence curve analysis

**Fig 9:**
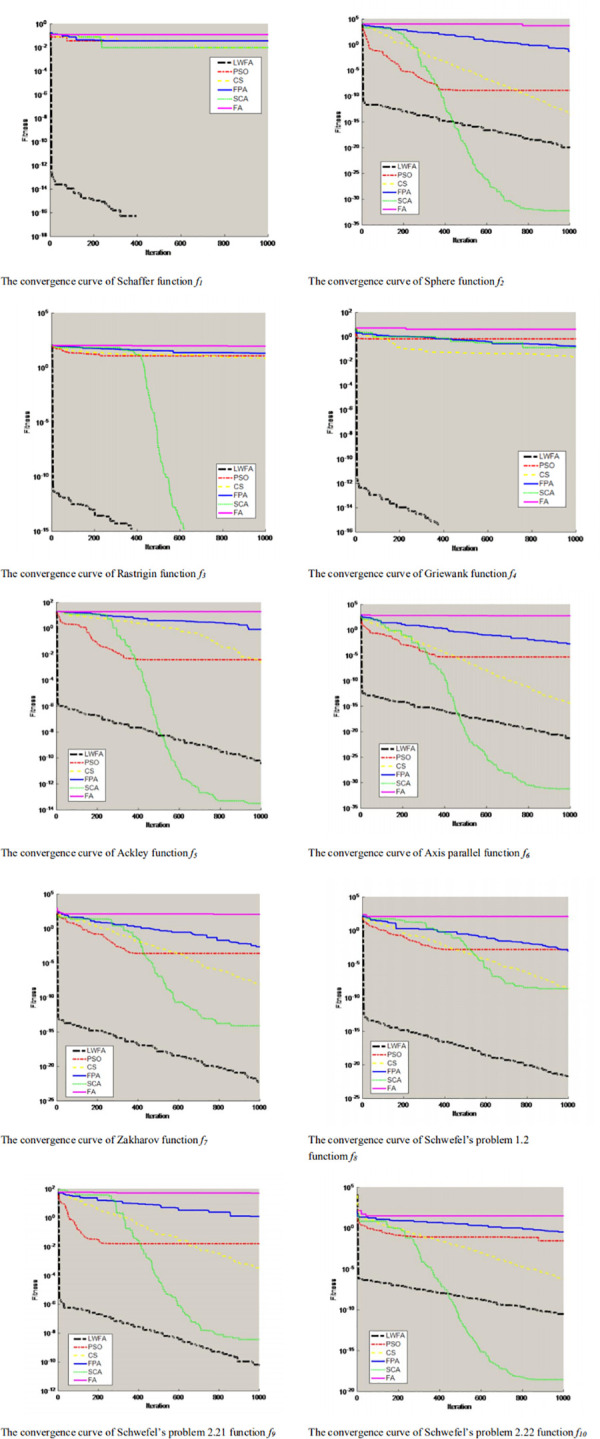
Convergence curve of functions *f*_*1*_*-f*_*10*_ in D = 10.

**Fig 10:**
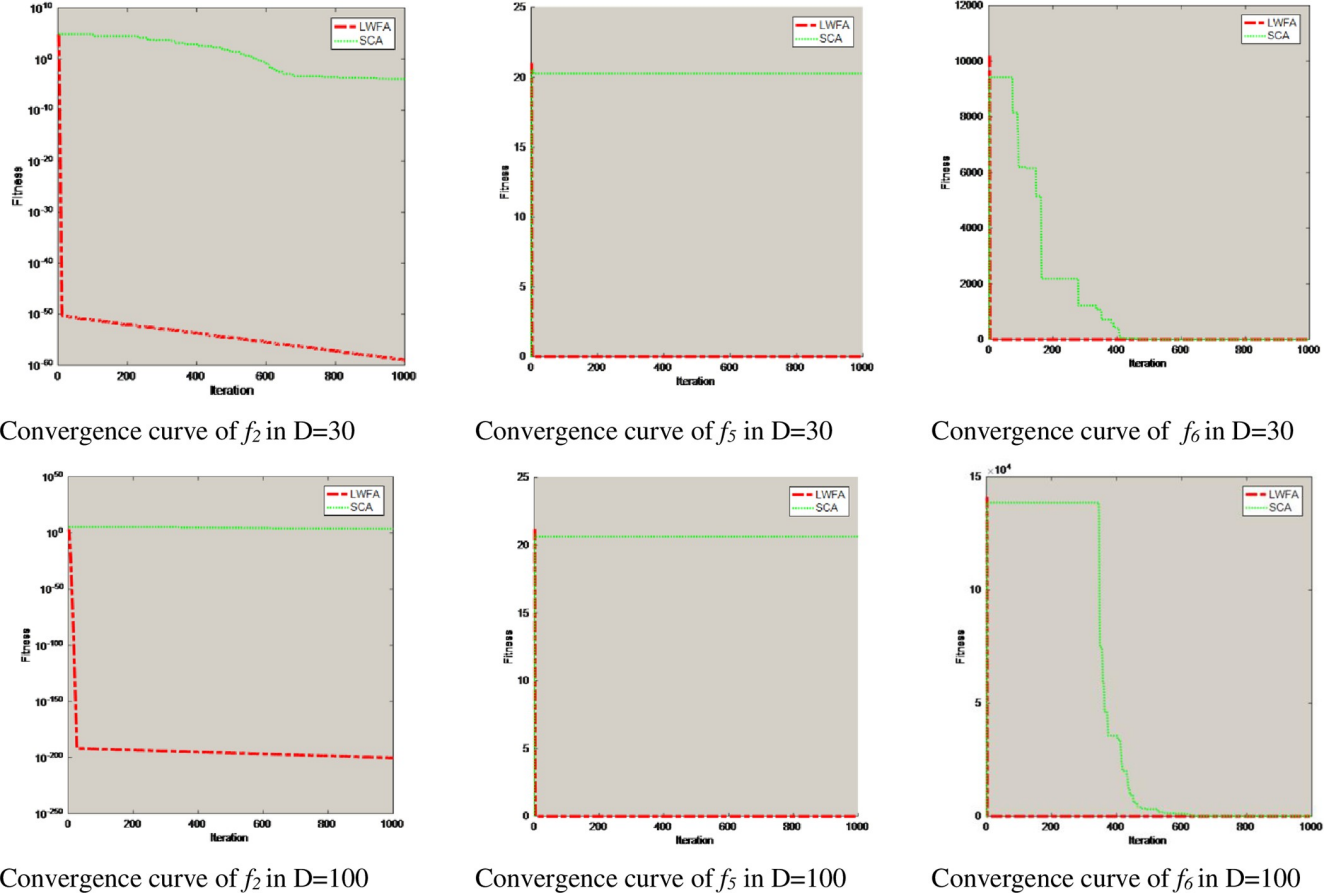
Contrast convergence curve of LWFA and SCA in D = 30/100.

To clearly show the convergence speed and accuracy of the LWFA, this paper will plot the convergence curves of the LWFA and the five other algorithms (PSO, the CS, FPA, SCA, and FA) with D = 10 on ten benchmark functions, as shown in Fig 9. When the dimension D = 30/100, the convergence curve of function *f*_*2*_, *f*_*5*_, and *f*_*6*_ are shown in Fig 10. In these graphs, the horizontal axes represent the iteration T = 1000, and the robustness of each algorithm is demonstrated in the vertical axes.

Fig 9 shows the performance of the LWFA. The other algorithm (PSO, the CS, FPA, and FA) have similar solutions based on the convergence curves. Compared with the SCA, the LWFA can obtain a higher accuracy for the function *f*_*2*_, *f*_*5*_, and *f*_*6*._ However, the performance of the SCA drops rapidly as the dimension increases. Fig 10 shows that the LWFA obtains a higher optimal value than the SCA.

Overall, based on the above comparison results above, we can see that the improved FA proposed in this paper is better than the other algorithms in the same dimension. When the comparison dimension is 30/100, the convergence rate of the LWFA is faster than the other algorithms in the convergence graph of the same function and has a vertical decline state. In different dimensions, the optimization accuracy of PSO, the CS, FPA, SCA, and FA will decrease with an increase in dimension. However, the optimization accuracy of the LWFA also increases gradually and shows good optimization performance for each function with an increase in its dimensions.

## 6 Conclusions

In this paper, the biological principle and mathematical description of the standard FA are presented, and the improvement of the FA and the research of its application fields are summarized. To achieve minimum attraction, an FA based on self-adaptive logarithmic inertia weight is proposed. The step adjustment factor is added to the random step term to dynamically adjust the random step, thus greatly improving the optimization performance of the FA. To evaluate the performance of the LWFA, multiple simulation experiments were conducted. In the first experiment set, ten benchmark test functions and IEEE CEC2010 standard test functions are used to compare the performance of state-of-the-art algorithms with that of the LWFA. The comparisons with PSO, the FPA, SCA, and CS show that the improved FA increases the accuracy of the solutions and significantly enhances the robustness of the solutions for the test functions, particularly for high-dimensional test functions. The experimental results in this study have convincingly shown and confirmed that the LWFA achieves high performance in solving optimization problems. The application of the improved FA to real-world engineering problems and multi-objective optimization will be done in future work.
